# Data Independent Acquisition Reveals In-Depth Serum Proteome Changes in Canine Leishmaniosis

**DOI:** 10.3390/metabo13030365

**Published:** 2023-02-28

**Authors:** Franjo Martinković, Marin Popović, Ozren Smolec, Vladimir Mrljak, Peter David Eckersall, Anita Horvatić

**Affiliations:** 1 Faculty of Veterinary Medicine, University of Zagreb, Heinzelova 55, HR-10000 Zagreb, Croatia; 2 Department of Safety and Protection, Karlovac University of Applied Sciences, Trg Josipa Juraja Strossmayera 9, HR-47000 Karlovac, Croatia; 3 School of Biodiversity, One Health and Veterinary Medicine, University of Glasgow, Bearsden Rd, Glasgow G61 1QH, UK; 4 Interdisciplinary Laboratory of Clinical Analysis of the University of Murcia (Interlab-UMU), Department of Animal Medicine and Surgery, Veterinary School, University of Murcia, 30100 Murcia, Spain; 5 Faculty of Food Technology and Biotechnology, University of Zagreb, Pierottijeva 6, HR-10000 Zagreb, Croatia

**Keywords:** biomarker discovery, canine, data independent acquisition, leishmaniosis, mass spectrometry, proteomics, serum

## Abstract

Comprehensive profiling of serum proteome provides valuable clues of health status and pathophysiological processes, making it the main strategy in biomarker discovery. However, the high dynamic range significantly decreases the number of detectable proteins, obstructing the insights into the underlying biological processes. To circumvent various serum enrichment methods, obtain high-quality proteome wide information using the next-generation proteomic, and study host response in canine leishmaniosis, we applied data-independent acquisition mass spectrometry (DIA-MS) for deep proteomic profiling of clinical samples. The non-depleted serum samples of healthy and naturally *Leishmania*-infected dogs were analyzed using the label-free 60-min gradient sequential window acquisition of all theoretical mass spectra (SWATH-MS) method. As a result, we identified 554 proteins, 140 of which differed significantly in abundance. Those were included in lipid metabolism, hematological abnormalities, immune response, and oxidative stress, providing valuable information about the complex molecular basis of the clinical and pathological landscape in canine leishmaniosis. Our results show that DIA-MS is a method of choice for understanding complex pathophysiological processes in serum and serum biomarker development.

## 1. Introduction

Blood serum reflects an individual’s phenotype providing a valuable information of physiological and pathological processes in organism making it a valuable source of biomarkers [1]. However, the high dynamic range aggravates the routine identification and quantification of low abundant proteins which are of a great interest to decipher proteome-wide protein functions and their interactions [2]. For that reason, various pre-analytical strategies such as fractionation, as well as enrichment methods including affinity depletion using immobilized antibodies or other molecules that specifically remove up to 22 high abundant proteins, and combinatorial peptide ligand library for proteome normalization are introduced [3,4]. Current depletion methods are costly, time-consuming, lack reproducibility, and often require additional processing (e.g., desalting or sample concentration) as a part of the MS-compatible proteomic analysis workflow. Additional separation steps are time consuming and introduce experimental variability making the results less reliable, especially regarding protein quantification [3]. Furthermore, most of the specific antibody-based depletion columns are constructed to remove the most abundant proteins from human samples unlike animal samples.

Mass spectrometry (MS)-based proteomics has become a powerful tool in biomedical research allowing the characterization of thousands of proteins as well as their posttranslational modifications [5]. Over the past couple of decades, MS-based proteomics has been mostly performed in a data-dependent acquisition (DDA) mode using shotgun strategy where proteins are digested by a sequence specific protease and resulting peptides are analyzed by liquid chromatography tandem mass spectrometry (LC- MS/MS) [4]. Herein, the top N most intense peptide’s fragments in MS2 produce a pattern of unique fragment ions spectra used to be assigned to their corresponding peptide sequences for unambiguous protein identification. However, low intensity ions often remain unidentified. Advances in MS-based technology and bioinformatics enabled to overcome the detectable dynamic range restrictions of peptides that ionize the best. Data-independent acquisition (DIA) using sequential window acquisition of all theoretical mass spectra (SWATH-MS) approach has been developed enabling the deep proteome coverage through confident peptide identification over a dynamic range of four orders of magnitude with quantitative consistency and accuracy [6]. The application of SWATH-MS enables the systematic and unbiased fragmentation of all ions within the overlapping precursor isolation windows leading to highly complex fragment ion spectra. Peptide identification is based on sample-specific spectral libraries constructed using DDA data, containing chromatographic and mass spectrometric coordinates determined by using normalized retention time [7]. Unlike DDA-based sample-specific spectral libraries, *in silico* spectral libraries are built directly from protein sequence databases (FASTA files) without previous DDA data requirements providing better proteome coverage in unbiased way [8]. For that purpose, deep learning algorithms are applied, selecting the list of target peptides from protein sequence databases by predicting the MS detectability of candidate proteotypic peptides [8]. It is worth mentioning that sample-specific spectral libraries (e.g., pan-human DIA libraries) are still the main choice in cancer research, reflecting tumor heterogeneity [9].

Zoonotic visceral leishmaniosis (VL) is a potentially fatal vector-borne disease endemic in South America, South-East Asia, Eastern Africa, and the Mediterranean Region [10]. In Croatia, human and canine leishmaniosis caused by *Leishmania infantum* has been present in central and southern Dalmatia since the beginning of the 20th century [11], and the dog has remained the main reservoir. Limited drug development and emerging drug resistance, as well as long duration of treatment [12] are making the understanding of the host-related processes in leishmaniosis even more important. In our previous studies, we applied tandem mass tag (TMT)-based DDA-MS methods for treatment monitoring in canine leishmaniosis by analyzing serum, and to understand the disease-related processes in canine saliva [11,12].

In that light, in order to obtain high-quality proteome wide information using the next-generation proteomics and study host response in canine leishmaniosis which could be observed in serum, the aim of this experiment was to construct canine serum proteome spectral library using DDA-MS approach and establish fast and label-free short gradient SWATH-MS method. Our results indicate the DIA-MS approach is extremely sensitive and informative, enabling the identification of canine serum proteins affected by canine leishmaniosis with biomarker potential, their functions and molecular pathways mostly included in lipid metabolism, hematological abnormalities, immune response, and oxidative stress.

## 2. Materials and Methods

### 2.1. Sample Description

The study was carried out using 10 archived canine sera previously used in routine leishmaniosis diagnostics at Department for Parasitology and Parasitic Diseases with Clinics, Faculty of Veterinary Medicine, University of Zagreb collected during six month- time interval. It involved sera classified into two groups: sera from healthy control group (N = 5) were mix breeds, age 2.5–9 years, while the *Leishmania*-infected group (N = 5) were mixed breeds, age 2–8 years. Control dogs were considered healthy based on previous serological tests. The infection of *Leishmania infantum* was diagnosed using three serological tests [13]: indirect fluorescence antibody test (IFAT), enzyme-linked immunosorbent assay (ELISA), and kinesin-related conserved recombinant antigen (rK39 rapid immunochromatographic test evaluating the presence of anti-*Leishmania* antibody) following the manufacturers’ instructions (Supplementary file S1). All naturally infected dogs (originating from the enzootic region of Croatia) showed clinical signs of *Leishmania* infection. The sera of infected dogs were taken before the treatment. All canine sera used in this study were tested negative for the presence of *Dirofilaria immitis* antigen using the antigen rapid test (Fast test HWantigen, Megacor Diagnostik, Vorarlberg, Austria). 

### 2.2. Sample Collection

Archived, 10 canine sera samples were received from different veterinary stations sent to Department for Parasitology and Parasitic Diseases with Clinics, Faculty of Veterinary Medicine, University of Zagreb, for routine serological leishmaniosis diagnostics. After the diagnostics, sera samples were aliquoted and stored at −80 °C until analyzed to avoid multiple freeze-thaw cycles. Total protein concentration in serum was determined using a Pierce BCA Protein Assay Kit (Thermo Scientific, Rockford, IL, USA).

### 2.3. Sample Preparation for Spectral Library Generation

#### 2.3.1. In-Solution Digestion

The pooled sample was prepared by mixing equal protein amounts of all ten samples involved in this study and used for in-solution digestion [14]. A total of 50 μg of total protein was mixed with 50 mM NH_4_HCO_3_ to a final concentration of 1 mg/mL. After the addition of 5 μL DTT (50 mM) sample was incubated for 30 min at 55 °C and 5 μL IAA (200 mM) was added subsequently. After 30 min, overnight digestion at 37 °C using trypsin gold (1:50 *w*/*w*) was performed. Tryptic digest was stored at −20 °C for further analysis [14]. Before the analysis, samples were mixed with 10× iRT peptide solution (Biognosys AG, Schlieren, Switzerland) for subsequent LC-MS/MS analysis.

#### 2.3.2. Strong Cation Exchange (SCX) Chromatography

SCX was performed using pipette tips [15] with some modifications. Before the SCX chromatography, digested sample was desalted using Cleanup C18 pipette tips (10 μL, Agilent Technologies, Santa Clara, CA, USA) according to manufacturer’s instructions. Finally, peptides were eluted with 80% ACN/0.1% (*v*/*v*) formic acid, dried using vacuum concentrator and dissolved in 10 μL 0.1% formic acid. Strong cation exchange chromatography was performed using OMIX SCX pipette tips (10 μL, Agilent Technologies, Santa Clara, CA, USA). The tips were washed by mixture of 25% ACN in 0.05% formic acid and peptides were subsequently eluted with 100, 200 and 400 mM NH_4_HCO_3_ in 25%ACN/0.05% formic acid (*v*/*v*). Finally, samples were dried in vacuum concentrator, desalted using Cleanup C18 pipette tips, dried again, and finally mixed with 18 μL loading buffer and 2 μL of 10× iRT peptide solution (Biognosys AG, Schlieren, Switzerland) for DDA analysis.

#### 2.3.3. Combinatorial Peptide Ligand Library (ProteoMiner)

The ProteoMiner Small-Capacity Kit (Bio-Rad, Hercules, CA, USA) containing a hexapeptide library [16] was used to equalize the protein concentration dynamic range of canine serum samples according to manufacturer’s procedure. Briefly, ProteoMiner column containing 20 μL beads was washed three times with 200 μL phosphate-buffered saline (PBS) buffer by 5 min incubations followed by 1000× *g* centrifugation. After adding 200 μL of pooled sample, bead-sample slurry was incubated with rotation (2 h at room temperature) and washed repeatedly three times before the elution. The protein elution was conducted by adding 20 µL of elution reagent to the column and vortexing for 15 min, followed by centrifugation at 1000× *g*.

As a part of MS-compatible workflow, eluted proteins were desalted using Microcon filter (10 kD MWCO, Millipore, Darmstadt, Germany). The eluate after ProteoMiner enrichment was mixed with 200 μL of milliQ water followed by centrifugation at 8000× *g* for 45 min. Proteins were washed twice with milliQ water by centrifugation at 8000× *g* for 30 min and once with 200 μL 50 mM NH_4_HCO_3._ Finally, proteins were collected by inverting the filter assembly followed by centrifugation at 2800× *g* for 5 min. Due to the reduced sample volume, the total protein concentration was determined by NanoDrop. Finally, in-solution digestion was performed. 

### 2.4. MS-Based Proteomic Analysis 

High resolution LC-MS/MS analysis was carried out using an Ultimate 3000 RSLCnano system (Dionex, Germering, Germany) coupled to a Q Exactive Plus mass spectrometer (Thermo Fisher Scientific, Bremen, Germany). Prior to analysis, tryptic peptides were mixed with iRT peptides (Biognosys) (1:10 *v*/*v*), desalted on the trap column and separated on the analytical column (PepMap™ RSLC C18, 50 cm × 75 μm). Ionization was achieved using nanospray Flex ion source (Thermo Fisher Scientific, Bremen, Germany) equipped with a 10 μm-inner diameter SilicaTip emitter (New Objective, Littleton, MA, USA).

#### DDA Proteomic Analysis for Spectral Library Generation

DDA analysis was performed as reported [14] with some changes. In short, for peptide separation, linear gradient 5–45% mobile phase B (0.1% formic acid in 80% ACN, *v*/*v*) over 120 min, at the flow rate of 300 nL/min was used. Mobile phase A contained 0.1% formic acid in water. The MS operated in positive ion mode using DDA Top10 method. Full scan MS spectra were acquired in range from *m/z* 350.0 to *m/z* 1800.0 with a resolution of 70,000, 120 ms injection time, AGC target 1 × 10^6^, a ±2.0 Da isolation window and the dynamic exclusion 30 s. For HCD fragmentation, step collision energy (29% and 35% NCE) with a resolution of 17,500 and AGC target of 2 × 10^5^ was used. Precursor ions with charge states of +1 and more than +7, as well as unassigned charge states, were excluded from HCD fragmentation. 

For DDA protein identification, the SEQUEST algorithm implemented into Proteome Discoverer (version 2.3., Thermo Fisher Scientific) was applied. Database search against database containing *Canis lupus familiaris* FASTA files (downloaded from UniprotKB database 14/10/2021, 49,889 entries) combined with Biognosys iRT fusion peptide FASTA file (available at https://biognosys.com/shop/iRT-Kit (accessed on 1 September 2022)) was performed using parameters as follows: two trypsin missed cleavage sites, precursor and fragment mass tolerances of 10 ppm and 0.02 Da, respectively; carbamidomethyl (C) fixed peptide modification, oxidation (M), dynamic modifications. The false discovery rate (FDR) for peptide identification was calculated by the Percolator algorithm within the Proteome Discoverer workflow and was set at 1%. 

The DDA mass spectrometry proteomics data have been deposited to the Consortium via the PRIDE partner repository [17] with the dataset PXD039763.

### 2.5. DIA Proteomic Analysis

For DIA analysis, a linear gradient 5–45% mobile phase B (0.1% formic acid in 80% ACN, *v*/*v*) over 60 min, at the flow rate of 300 nL/min was used. Mobile phase A contained 0.1% formic acid in water. The MS operated in positive ion mode using DIA Full MS method. MS spectra representing 44 overlapping sequential DIA windows with an isolation width of *m/z* 15 between *m/z* 450 and *m/z* 1100 were acquired using resolution of 35,000 and AGC target 1 × 10^6^, MSX 1, and charge state +2. For HCD, fragmentation collision energy 29% (NCE) with a resolution of 17,500 and AGC target of 2 × 10^5^ was used. The raw and analyzed DIA data files have been deposited to the ProteomeXchange Consortium via the PRIDE partner repository [17] with the dataset PXD039765.

Obtained data were analyzed using Spectronaut 16 (v. 16.2.220203.530000, Biognosys AG, Schlieren, Switzerland) [18]. Protein identification and quantification was performed using directDIA workflow. Identification was improved by combining of in-house generated spectral library and FASTA files (as described in Section 2.5). Factory settings were used to perform differential expression analysis between two different conditions, e.g., healthy and *Leishmania*-infected dogs and calculate adjusted *p*-values (q-values) based on multiple hypotheses testing corrections using Benjamini–Hochberg method. Proteins having adjusted *p*-value (q-value) < 0.05 were considered as significantly abundant. Principal component analysis (PCA) plot and volcano plot were generated in Spectronaut software. Gene ontology (GO) terms were enriched based on Welch’s t-test with *p*-value < 0.05 using bioinformatics web platform ExpressAnalyst (https://www.expressanalyst.ca/ (accessed on 30 November 2022)). For the network-based and enrichment analyses, *Homo sapiens* was selected as model organism. 

### 2.6. Spectral Library Generation

For spectral libraries generation, DDA raw files and belonging Proteome Discoverer search results of analyzed samples (namely pooled, mixed control, mixed case, ProteoMiner enriched, and 3 SCX fractions all containing iRT peptides for retention time normalization as described in Section 2.3) were imported into Spectronaut 16 software (v. 16.2.220203.530000, Biognosys AG, Schlieren, Switzerland) with default settings. Furthermore, to enable *in silico* spectral library generation using *Canis lupus familiaris* FASTA files downloaded from UniprotKB database 14/10/2021, 49,889 entries) combined with Biognosys iRT fusion peptide FASTA file (available at https://biognosys.com/shop/iRT-Kit (accessed on 1 September 2022)), Pulsar search engine with deep learning augmentation integrated into Spectronaut software was used. For libraries generation, following factory settings were used: Trypsin/P as the digestion enzyme with missed cleavages ≤2, and only peptides with length from 7 to 50 amino acids with mass ≤ 6000 Da were kept. 

## 3. Results

### 3.1. DDA-Based Proteomic Results

**Figure 1:**
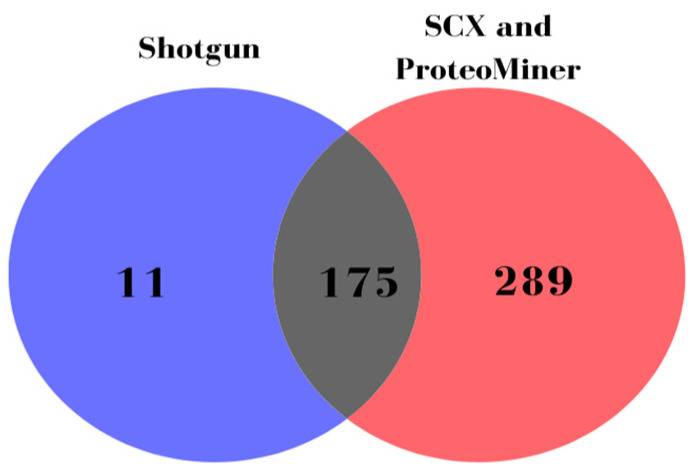
DDA-MS analysis reveals increase in number of identified serum protein groups (master proteins) after enrichment and fractionation (ProteoMiner and SCX fractions) compared to non-depleted sample without fractionation (Shotgun).

To generate high-quality sample-specific spectral library containing a large set of high confident peptides originating from both high and low abundant serum proteins, a pooled and ProteoMiner enriched samples as well as SCX fractions were analyzed by DDA-MS-based strategy. In this way, we identified 1405 proteins within 464 protein groups (464 master proteins), unlike 186 protein groups identified only by analyzing the pooled sample (shotgun approach without depletion and fractionation), as shown in Figure 1. 

### 3.2. DIA-Based Proteomic Results

**Figure 2:**
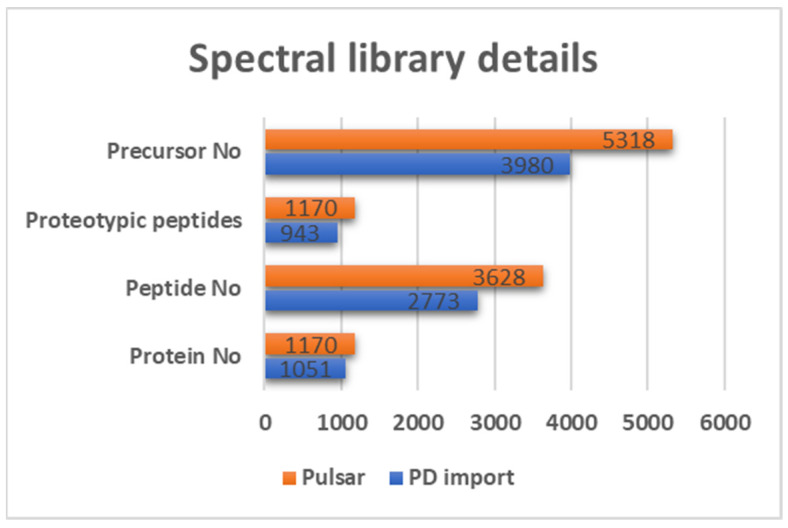
Spectral libraries generated by Spectronaut 16 Pulsar database search (in orange) and sample-specific library generated by Proteome Discoverer import (in blue). Both spectral libraries are provided through the Supplementary file S2.

DIA-based proteomic analysis was employed to provide the deep insight into proteome-wide changes of host serum in canine leishmaniosis. For generating state-of-the-art libraries from both DDA data, Pulsar search integrated into Spectronaut software was used. The sample-specific spectral library (provided in Supplementary file S2) contained *Canis lupus familiaris* FASTA file enriched with peptides identified by DDA analysis, e.g., pooled, enriched and/or fractionated serum samples (Pulsar search). Spectral library details are shown in Figure 2. 

**Figure 3:**
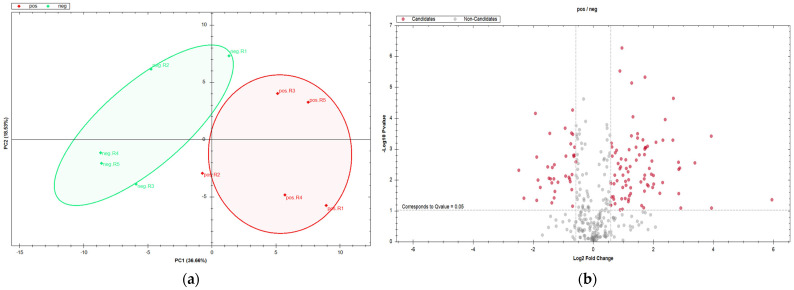
(**a**) Principal component analysis (PCA) score plot of the second and first principal components (PC1 vs PC2) reveals the differences in serum proteomes from healthy (neg, in green) and *Leishmania*-infected dogs (pos, in red). (**b**) Volcano plot of DIA data depicted with the fold change of each differentially abundant protein and belonging q-value. Significantly differentially abundant proteins (q < 0.05) are highlighted in red (marked as Candidates).

Quantitative proteomic data of non-depleted serum samples were obtained using the SWATH-MS technology as described in Section 2.5. DIA proteomic analysis. As a result, we successfully identified a set of 554 serum proteins with a high quantitative performance. Furthermore, proteomic analysis with a wide dynamic range coverage revealed difference in the abundance of the 140 proteins with 107 being higher and 33 lower in abundance in canine leishmaniosis satisfying the cutoff criteria (q-value < 0.05 and absolute log2FC > 0.5). Belonging volcano plot is depicted in Figure 3b. The principal component analysis (PCA) plot shows differences in serum proteomes between healthy and *Leishmania*-infected dogs as in Figure 3a. 

A shortened list containing differentially abundant proteins that change the most (the absolute value) and accompanying details, such as accession numbers and fold changes, is provided within the Table 1. The complete protein list can be found within Supplementary file S3.

**Figure 4:**
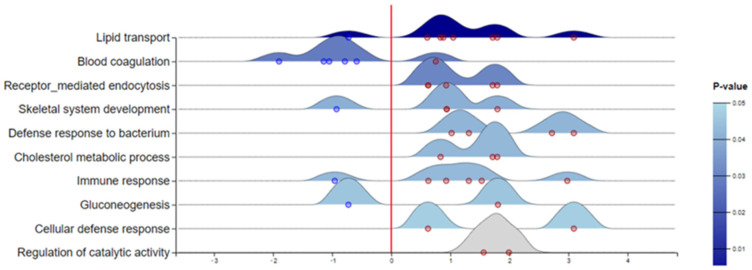
Ridgeline diagram of enriched GO (database PANTHER: BP) showing the biological processes and log2FC of belonging deregulated proteins (red dots—up; blue dots—down) in serum of dogs with leishmaniosis. The shade of blue represents *p*-values as depicted. BP in grey is not significant. The GO diagram is created using ExpressAnalyst (https://www.expressanalyst.ca/ (accessed on 30 November 2022)) based on Welch’s *t*-test.

**Figure 5:**
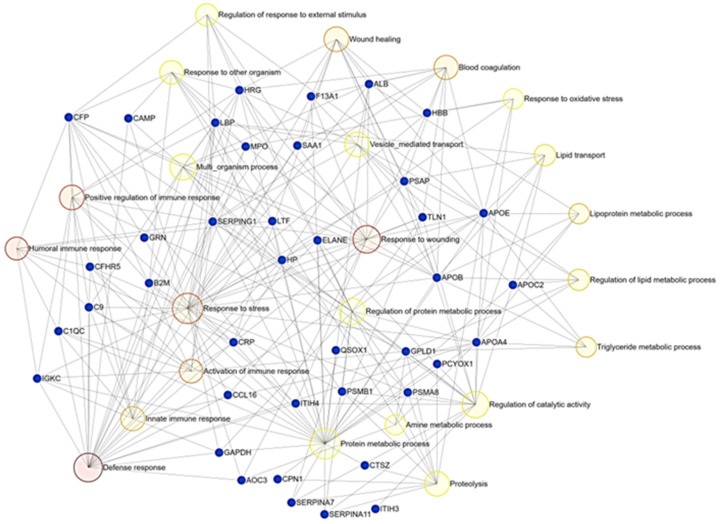
Bipartite network showing the results of enrichment analysis of deregulated genes (GO:MF, based on adjusted *p*-values ranked by Welch’s test) in serum of *Leishmania*-infected dogs compared to healthy ones performed using ExpressAnalyst (https://www.expressanalyst.ca/ (accessed on 30 November 2022)). Blue dots represent proteins, while yellow/red circles mark molecular functions affected by canine leishmaniosis.

Gene ontology (GO) analysis of differentially abundant proteins revealed key molecular functions and biological processes affected by *Leishmania* infection (Figure 4 and Figure 5). The GO term results (Figure 4) indicate that the proteins affected by *Leishmania* infection play roles in lipid transport (*p*-value 2.08 × 10^−7^) and cholesterol metabolism (*p*-value 8.3 × 10^−3^), blood coagulation (*p*-value 2.7 × 10^−3^), immune response (*p*-value 9.2 × 10^−3^), and receptor-mediated endocytosis (*p*-value 4.7 × 10^−4^), among others.

## 4. Discussion

Serum proteomics is a powerful tool for non-invasive monitoring of biological processes, enabling the understanding of various pathophysiological processes occurring in a living organism as well as biomarker discovery. However, although easily accessible, high abundant molecules, such as albumin (57–71%) or gamma-globulins (8–26%), as well as high dynamic range (12 orders of magnitude) molecules, have been making the serum a complex analyte for DDA bottom-up MS analysis, unlike our DIA-MS study where 554 proteins were identified in non-depleted sample. Although various depletion strategies of high abundant proteins have been introduced, serum albumin is a 66.5 kDa-protein known to act as a carrier for various endogenous and exogenous ligands [19] and other small proteins [20]. So, the removal of these large molecules often results in the loss of molecules of interest (in terms of quantity and/or quality) providing an incomplete picture. Aware of the depletion effect on the results, in our previous studies, we analyzed non-depleted serum samples in dogs with leishmaniosis for treatment monitoring using DDA tandem mass tag (TMT) label-based quantitative proteomic approach which enabled us the identification of 117 canine serum proteins among which 23 were differentially abundant [21]. However, based on the number of total identified proteins, the detection of low abundant proteins remained partially disabled, still giving the incomplete insight into the complex serum proteome. Despite DDA-MS analysis was the main choice in proteomics since its inception, DIA-based proteomics experiments are recently becoming the powerful alternative due to the fast and simplified sample preparation and significantly improved results in terms of reduced instrument time requirements and the increased number of identified proteins [22], which we also showed herein. Moreover, results addressing DIA-MS-based plasma proteomics in dogs have been reported confirming all abovementioned methodological advantages [23]. However, DIA has not yet been applied in the analysis of sera in canine leishmaniosis. Herein, we aimed to implement DIA serum proteomics workflow using next generation proteomic technology to better understand the host-specific processes during *Leishmania* infection in dogs. 

For in-depth DIA-MS proteome analysis, we established high-quality sample specific spectral library containing extensive set of 3628 peptides and their relative retention time details obtained by spiked-in non-naturally occurring synthetic peptides for retention time normalization [7] to enable proteome profiling coverage. Having in mind that the spectral library should be sufficiently large, sample-size comparable, and that the results are library (size and fragmentation pattern) dependent [24], we chose the directDIA workflow based on *in silico* database search (canine FASTA files) enriched with Pulsar search results of our DDA data for the best result outputs. In this way, using SWATH methodology, we identified 554 serum proteins using 60-min gradient time, which is about 4.5 times more protein than in our previous study [8] or about 2.5 times more proteins than performing the DDA without depletion (120-min gradient time). This enabled detailed qualitative and quantitative insights into the serum proteomes of healthy and *Leishmania*-infected dogs, including both high and low abundant proteins with simplified sample preparation and shorter analysis time. Although the DIA database search was performed, *Leishmania* proteins were not found (data not shown). It is important to emphasize that some of the leishmaniosis-related serum proteins (such as prosaposin) identified herein by simple sample preparation (in-solution digestion) and SWATH-MS proteomic approach were previously identified only after laborious sample preparation (exosome isolation) and subsequent proteomic analysis [25]. Finally, GO analysis of differentially abundant proteins provided us key molecules and related molecular processes observable in serum affected by *Leishmania* infection in dogs. Those include lipid metabolism, hematological abnormalities, immune response, oxidative stress, etc. 

### 4.1. Lipid Metabolism and Transport

*Leishmania infantum* is an intracellular parasite which relies on host lipid reservoir and synthesis mechanism for its survival [26]. Various studies related to *Leishmania* infection reported the multiple importance of cholesterol and cholesterol metabolic processes in *Leishmania* transformation process and sterol metabolism [27]. The cholesterol sequestration by *Leishmania* parasite begins post infection, with host cells aiming to restore initial cholesterol levels correlating with the upregulation of proteins required for cholesterol metabolism [28]. Accordingly, in our study, cholesterol metabolic processes are shown to be affected by *Leishmania* infection, with proteins Apo E and Apo A4 being upregulated. The elevated levels of Apo E have already been reported in serum of *Leishmania*-infected human patients [29]. Apo E (apolipoprotein E) is a 34 kDa glycoprotein critical for cholesterol transport and lipoprotein particle metabolism, playing an important role in lipolytic enzyme activation and immune response [30]. The study involving apolipoprotein E knockout mice infected with *Leishmania donovani* showed the important role of Apo E in protection against visceral leishmaniosis by displaying hypercholesterolemia, host-protective cytokines, and expansion of antileishmanial CD8 + IFN-γ + and CD8 + IFN-γ +TNF-α + T cells [31]. Apolipoprotein A4 (APOA4) is a plasma lipoprotein involved in the regulation of lipid and glucose metabolism [32], and enhances triglyceride (TG) secretion from the liver [33]. Upregulation of APOA4 protein shown herein could be related with already reported increase in triglyceride levels in patients with visceral leishmaniosis [34]. 

Except cholesterol, the studies have been published indicating that *Leishmania* sp. scavenges host sphingolipids (e.g., sphingomyelin, which is not synthesized by *Leishmania* but is abundant in mammals) needed for amastigote proliferation, virulence and biosynthesis of inositol phosphorylceramide (IPC, major sphingolipid in the genus *Leishmania* not present in mammals) [35]. In our study, we showed the upregulation of prosaposin, PSAP, which is a large precursor protein that is proteolytically cleaved into four sphingolipid activator proteins that assist in the lysosomal hydrolysis of sphingolipids into ceramide needed for the synthesis of IPC [36,37]. Our results are in accordance with existing data showing the *Leishmania* infection induces the host ceramide synthesis pathway [38].

### 4.2. Hematological Abnormalities

The progression of the canine leishmaniosis has the effect on hematological parameters, mostly caused by anemia and/or platelet aggregation abnormalities [39]. Our results are supporting these findings. Anemia is present in the majority of symptomatic dogs with leishmaniosis [39]. In our study, we detected the decreased amount of carbonic anhydrase 1 (CA1). CA1 is the most abundant protein in erythrocytes after hemoglobin. Its expression increases in various forms of anemia. However, its expression decreases in hemolytic anemia and for that reason CA1 is used as biomarker for this disease [40]. 

Coagulation factors, being a part of coagulation cascade, are essential for normal blood clotting. Herein, we determined the relative coagulation factor (F11, F13A1, F13B) deficiency, as well as histidine-rich glycoprotein (HRG) deficiency in *Leishmania*-infected dogs compared to healthy dogs that is related to decreased coagulation factor activities and fibrinolysis as reported in human patients [41,42,43]. Furthermore, we also found platelet-activating factor (PAF) acetylhydrolase (PLA-AH2) to be upregulated in *Leishmania*-infected dogs. Platelet-activating factor is a phospholipid that is involved in the activation of thrombotic cascades, induces leukopenia and thrombocytopenia (which are the most common hematological symptoms in leishmaniosis), as well as increases vascular permeability [44]. It is also involved in inflammatory reactions. PAF acetylhydrolase 2, also known as lipoprotein-associated phospholipase A2 (Lp-PLA2), inactivates the PAF and PAF-like oxidized phospholipids found in oxidized LDL by hydrolysis, controlling their actions. The increase in PLA-AH2 activity is positively correlated with activation of inflammatory cells. PAF was reported essential for the control of *Leishmania* infection. [45].

### 4.3. Immune Response

The immune response is a coordinated response of variety of cells and molecules on exposure to foreign antigens. Acute phase proteins (APPs) are the markers of the immune system response whose concentration in serum significantly changes during inflammation, which was also found in our study. The APP profiles and concentrations in combination with animal clinical condition monitored at physical examination may provide a valuable tool for the characterization, management and treatment monitoring of canine leishmaniosis [46]. Canine leishmaniosis is related with the increase of haptoglobin (HB) and C-reactive protein (CRP, or pentraxin) concentration that is measured in clinical practice [47]. Except increase in concentration of HB, CRP, and alpha 2 macroglobulin (A2M) in serum of *Leishmania*-infected dogs, we also detected negative APPs, transthyretin and retinol-binding protein, showing the decrease in abundance in *Leishmania*-infected dogs compared to healthy dogs. Interestingly, alpha 1 antitrypsin (ATT), a positive APP, was found to be lowered in *Leishmania*-infected dogs. Alpha-1 antitrypsin is a protease inhibitor synthesized mainly in the liver. Diffusing into the lung tissue and alveolar fluid, it inactivates neutrophil elastase and protects the lung tissue from the damage. The reduction of circulating levels of alpha-1 antitrypsin result in the increased risk of lung disease [48]. Accordingly, the pulmonary alterations and the inflammatory process defined as interstitial pneumonitis were reported in *Leishmania-*infected dogs, as well as the presence of amastigotes in canine lungs, which could support our findings [49]. 

Except the APPS, within the immune response, lysosomal cysteine cathepsin family of proteases, such as cathepsin S (CTSS), play a key role. CTSS, involved in TLR9 processing [50], is involved in dendritic cells activation and resolution of *Leishmania major* infection in mice [51]. Also, cathepsin S deficient mice are slightly more susceptible against *Leishmania major* infection [52]. According to those facts, animals with elevated cathepsin S in serum should be resistant against infection with *Leishmania*, which is not the case in our work. In contrast, in our survey, the amount of cathepsin S was three-fold elevated in positive dogs. As elevated cathepsin S is proven in many human diseases [53,54], a more detailed investigation should be made to show if the cathepsin S is a good marker for the disease diagnostics and prognosis.

Although the DIA-SWATH-MS strategy applied herein generated a long list of deregulated proteins, some of which present biomarker potential, further investigation using a larger population of healthy and *Leishmania*-infected canine patients is needed to determine the diagnostic potential of a single biomarker or biomarker panels, correlating the clinical symptoms and protein concentration. Additionally, it would be interesting to use a DIA-MS approach for canine leishmaniosis treatment monitoring.

## 5. Conclusions

Our study demonstrates the advantages of next generation DIA-MS proteomic analysis of serum, enabling deeper insights into altered expression levels of both high and low abundant proteins without enrichment in shorter analysis times. This approach was shown to be extremely useful and informative in the identification of disease-related proteins, their functions, and molecular pathways in canine leishmaniosis. Additionally, our open access canine serum spectral libraries could be useful for *in silico* generated combined spectral libraries to be applied in various canine serum studies. Finally, this approach may be employed to decipher the pathological processes of various diseases in serum, as well as serum biomarker development. 

## Figures and Tables

**Table 1:** The shortened list of differentially abundant proteins in *Leishmania*-infected vs healthy dogs obtained by directDIA MS analysis in Spectronaut 16 software.

Uniprot Accession ID	Protein Name	Gene Name	Log2FC	q-Value *
F1PP29	Uncharacterized protein	ND **	−2.60	0.00423
A0A5F4CGT3;F1PWW0	Filamin A	FLNA	−2.42	0.02513
Q6JDI3	Plasminogen (Fragment)	PLG	−1.90	0.00002
F1PBK6	Carbonic anhydrase	CA1	−1.88	0.01058
E2R5U8	Transthyretin	TTR	−1.56	0.03280
E2R1N7	Prenylcysteine oxidase 1	PCYOX1	−1.51	0.00106
F1PCG4	Peroxiredoxin 2	PRDX2	−1.49	0.00102
E2RT38;F1PAQ3	Maltase-glucoamylase	MGAM	−1.47	0.00048
A0A5F4D5S2;J9PAD1	C4a anaphylatoxin	LOC481722	−1.41	0.00000
F1PZC6	Histidine rich glycoprotein	HRG	−1.14	0.00139
A0A5F4C5S3;F1PNR5	Coagulation factor XI	F11	−1.05	0.00722
E2RA67	C-C motif chemokine	CCL14	−0.96	0.00516
F1PD34	Secreted phosphoprotein 2	SPP2	−0.93	0.00371
A1ILJ0	Alpha 1 antitrypsin	SERPINA1	−0.92	0.01290
A0A5F4CEP4	Fetuin B	FETUB	−0.90	0.00197
A0A5F4D9Z3;F1PB85	Serpin family A member 7	SERPINA7	−0.88	0.03724
D2K841;J9P346	C-X-C motif chemokine;	PPBP	−0.87	0.00888
E2RPW3	Paraoxonase	PON3	−0.85	0.00029
A0A5F4BY85;A0A5F4CHL2; E2RK02	Glycosyl-phosphatidylinositol-specific phospholipase D	GPLD1	−0.84	0.00129
A0A5F4CZV0;F2Z4P4;J9P9I7	Elongation factor 1-alpha	LOC102155289	−0.83	0.02608
A0A5F4DCK5;F1PKX3	Coagulation factor XIII A chain	F13A1	−0.78	0.04243
Q28275-1	Isoform 1 of Fibronectin	FN1	−0.77	0.00018
A0A5F4CT11;E2RE97	Numb-like protein	COQ8B	−0.77	0.00044
A0A5F4CD78;A0A5F4DFV6;E2R3V1	Fc receptor like 4	FCRL4	−0.74	0.03556
A0A5F4CED7;F1Q4D9	Plasma retinol-binding protein	RBP4	−0.73	0.01947
F1P8Z5	Apolipoprotein B	APOB	1.72	0.00011
F1PNY2	Immunoglobulin kappa	IGKC	1.73	0.00066
A0A5F4D392;A0A5F4D5Q6;J9NX46	Clathrin light chain A	CLTA	1.73	0.01765
Q2EG92	Lubricin (Fragment)	PRG4	1.79	0.00138
A0A5S7EUL7;P18649	Apolipoprotein E	APOE	1.79	0.00194
A0A5F4BSV4;A0A5F4C284;A0A5F4C8H6;A0A5F4CPB8;F1PIA3	Fibrillin 1	FBN1	1.80	0.02898
F1PBT3;J9P7A6	Fructose-bisphosphate aldolase	ALDOA	1.81	0.00606
A0A5F4CYW8;E2R6Q7	Cathepsin B	CTSB	1.99	0.00071
A0A5F4C3X0;A0A5F4CAZ5;E2QY07	Actinin alpha 1	ACTN1	2.05	0.04209
F1PKL6;L7N0K1	Ig-like domain-containing protein	ND	2.44	0.00506
E2R5J0;T2KEN6	Pentaxin	CRP	2.45	0.00301
J9NT12	Fibrinogen like 1	FGL1	2.47	0.01586
A0A5F4D8N3;A0A5F4DDP8	Lymphocyte cytosolic protein 1	LCP1	2.50	0.00036
E2RJY0	Potassium channel tetramerization domain containing 12	KCTD12	2.67	0.01294
F1PQ52	Myeloperoxidase	MPO	2.72	0.01008
E2RHN1	Granulin precursor	GRN	2.84	0.00137
A0A5F4C236;A0A5F4CUH8;A0A5F4DFI4;E2RLA5	Golgi membrane protein 1	GOLM1	2.92	0.00622
F1PAK0;Q8HY81	Cathepsin S	CTSS	2.98	0.00043
E2QW61	Lipopolysaccharide-binding protein	LBP	3.09	0.00238
E2R8C5	Immunoglobulin lambda variable 2-33	IGLV2-33	4.04	0.00158

* adjusted *p*-values (Benjamini–Hochberg method); ** not defined.

## Data Availability

The DDA mass spectrometry proteomics data have been deposited to the Consortium via the PRIDE partner repository with the dataset PXD039763. The DIA-MS data files have been deposited to the ProteomeXchange Consortium via the PRIDE partner repository with the dataset PXD039765.

## Supplementary Materials

The following supporting information can be downloaded at: https://www.mdpi.com/article/10.3390/metabo13030365/s1, Supplementary file S1. Diagnostic methods for determination of Leishmania infection in selected canine serum samples. The infection of *Leishmania infantum* was diagnosed using three serological tests: indirect fluorescence antibody test (IFAT), enzyme-linked immunosorbent assay (ELISA) and kinesin-related conserved recombinant antigen (rK39 rapid immunochromatographic test evaluating the presence of anti-*Leishmania* antibody, Supplementary file S2. Spectral libraries generated by Spectronaut 16 Pulsar database search and sample-specific library generated by Proteome Discoverer import in Spectronaut 16. Supplementary file S3. Differentially abundant proteins detected in serum of *Leishmania*-infected dogs compared to healthy dogs by employing DIA-MS.

## List of Abbreviations

A2M	alpha 2 macroglobulin
ACN	acetonitrile
AGC	automatic gain control
Apo A4	apolipoprotein A4
Apo E	apolipoprotein E
APP	acute phase protein
ATT	alpha 1 antitrypsin
BCA	bicinchoninic acid
BP	biological process
CA1	carbonic anhydrase 1
CD8+ T cells	cytotoxic T lymphocytes
CRP	C-reactive protein, or pentraxin
CTSS	cathepsin S
DDA	data-dependent acquisition
DIA-MS	data-independent acquisition mass spectrometry
DTT	dithiothreitol
ELISA	enzyme-linked immunosorbent assay
FC	fold change
FDR	false discovery rate
GO	gene ontology
HB	haptoglobin
HCD	higher energy collisional dissociation
HRG	histidine-rich glycoprotein
IAA	iodoacetamide
IFAT	indirect fluorescence antibody test
IFN-γ	interferon gamma
IPC	inositol phosphorylceramide
iRT	indexed retention time
LC-MS/MS	liquid chromatography tandem mass spectrometry
LDL	low density lipoprotein
Lp-PLA2	lipoprotein-associated phospholipase A2
MF	molecular function
MWCO	moleculat weight cut off
NCE	normalized collision energy
PAF	platelet-activating factor
PCA	principal component analysis
PSAP	prosaposin
rK39	kinesin-related conserved recombinant antigen
SCX	strong cation exchange
SWATH	sequential window acquisition of all theoretical mass spectra
TG	triglyceride
TLR9	toll-like receptor 9
TMT	tandem mass tag
TNF-α	tumor necrosis factor alpha
