# Supplementary material for: Data Independent Acquisition Reveals In-Depth Serum Proteome Changes in Canine Leishmaniosis
Source: Metabolites. 2023 Feb 28;13(3):365. doi: 10.3390/metabo13030365 (PMC10059658; doi:10.3390/metabo13030365)
Supplement: Supplementary file 1 [file metabolites-13-00365-s001.zip › Supplementary file S1.pdf]

Supplementary Table S1. Diagnostic methods for determination of *Leishmania* infection in selected canine serum samples. The infection of *Leishmania infantum* was diagnosed using three serological tests: indirect fluorescence antibody test (IFAT), enzyme-linked immunosorbent assay (ELISA) and kinesin-related conserved recombinant antigen (rK39 rapid immunochromatographic test evaluating the presence of anti-*Leishmania* antibody).

Sample	Animal data	Diagnostic method		
		**IFAT**	**ELISA**	**Other**
2274	Dogo argentino, 2.5y., m., VEGAS	1/1280 pos	pos	rK39 (+)
2282	staffordshire terrier, 3y., m., DE SIMBA	1/640 pos	pos	rK39 (+)
2287	mixed breed, 2.5y., m., DŽAT	1/640 pos	pos	rK39 (+)
2304	german shepherd, 9y., m., MAX	1/640 pos	pos	rK39 (+)
2315	german shepherd, 2y., m., TIMBO	1/2560 pos	pos	rK39 (+)
2289	mixed breed, 2y., m., GARO	1/40 neg	neg	rK39 (-)
2303	american staffordshire terrier, 8y., m., BUBI	1/40 neg	neg	rK39 (-)
2311	mixed breed, 4y., f., BETTY	1/40 neg	neg	rK39 (-)
2317	mixed breed, 5y, m., ŽUĆO	1/40 neg	neg	rK39 (-)
2318	mixed breed, 6y., f., BELA	1/40 neg	neg	rK39 (-)
